# Bilirubin-induced neurotoxicity and visuocortical dysfunction

**DOI:** 10.1038/s41372-022-01417-2

**Published:** 2022-05-26

**Authors:** William V. Good, Ronald J. Wong, Anthony M. Norcia, David K. Stevenson, Terri Slagel, Chuan Hou, Vinod K. Bhutani

**Affiliations:** 1 Smith-Kettlewell Eye Research Institute, San Francisco, CA USA; 2 California Pacific Medical Center, Department of Pediatrics, San Francisco, CA USA; 3 Department of Pediatrics, Division of Neonatal and Developmental Medicine, Stanford University School of Medicine, Stanford, CA 94305 USA; 4 Department of Psychology, Wu Tsai Neurosciences Institute, Stanford University, Stanford, CA 94305 USA

**Keywords:** Disease model, Outcomes research, Risk factors, Translational research

Bilirubin-induced neurologic dysfunction (BIND) attributed to excessive bilirubin production and isoimmunization is probably amenable to prevention by early identification of hemolysis and blood group incompatibilities as well as timely, effective interventions such as phototherapy [1, 2]. Nevertheless, precise intervention thresholds for the most vulnerable infants remain elusive. Clinical reliance on total serum/plasma bilirubin (TB) or levels has not yet been complemented with accurate measurements of bilirubin-binding and unbound bilirubin (UB). It is the latter that has been implicated in widespread neurological dysfunction, characterized as the syndrome of BIND [3]. Of these, disturbances of visuo-oculomotor, auditory, speech, cognition, and language among children have been proposed [3, 4]. Perturbations of infant visuocortical development, assessed by serial contrast sensitivity and vernier acuity measurements using sweep visual evoke potentials (sVEPs) have been implicated in long-term consequences of bilirubin exposure, including impaired visual acuity [4].

Here, we report that neonatal hyperbilirubinemia causes significant, long-lasting negative effects on the developing *visual cortex*, even at TB levels not considered harmful. We explored whether less profound but significant effects on visual acuity occurs during infancy at lower total bilirubin (as measured by TB or transcutaneous bilirubin [TcB]) levels, and whether there is an overall correlation with severity of hyperbilirubinemia. This observational study showed that BIND can affect the visual cortex with impairments failing to improve with time through the first year after birth. The methods used in this study demonstrate that BIND may be associated with long-term visual disorders, at least including a reduction in visual acuity. Other disorders of vision are possible, but require additional follow up.

**Fig. 1:**
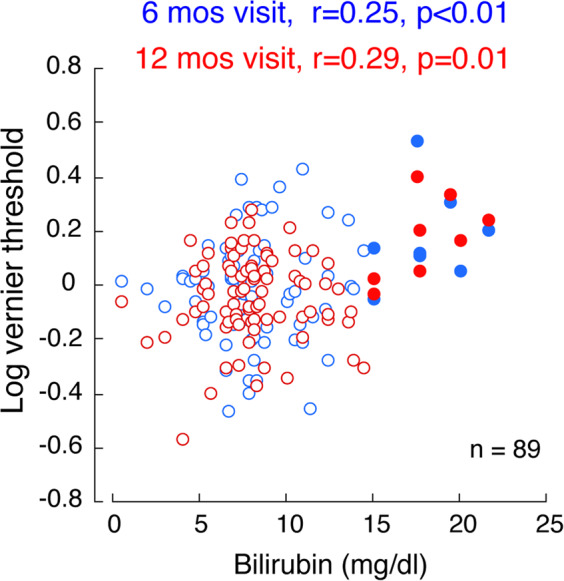
Correlation between vernier acuity and bilirubin (TB) levels. The blue and red circles represent vernier acuity thresholds at 6 and at 12 months of age, respectively. The solid circles represent infants with TB levels above 15 mg/dl. Correlation coefficients and significances were calculated using one-tailed Pearson’s R.

We followed 89 consecutive full-term healthy infants to 12 months of age. Peak total bilirubin levels were determined by serial TB or TcB measurements and ranged from 2 to 22.9 mg/dL at admission. At 6 and 12 months of age, specific cortical function (contrast sensitivity, vernier acuity, and grating acuity) was assessed using sVEPs [4]. These 3 functions reflect the integrity of different visuocortical mechanisms. We found a significant correlation between peak total bilirubin levels and vernier acuity thresholds (see Fig. 1) and contrast thresholds, with worsening at higher levels of bilirubin. Grating acuity did not correlate with bilirubin levels. These findings were unchanged whether TB or TcB was used alone or with UB. The solid circles in the figure are children in the top quartile on the hour-specific bilirubin nomogram. Thresholds are expressed in log units on the Y-axis. Higher levels on the axis indicate worse acuity. The X-axis shows the total bilirubin (serum or, TcB) measures shortly after birth. Blue circles represent vernier thresholds at 6 months of age; red circles at 12 months of age in the same group of children. Similar findings occurred with contrast sensitivity acuity.

Importantly, these finding persisted to 12 months of age suggesting that alterations in visuocortical function endure through at least the first year after birth. The clinical significance of these findings is as yet unknown, but are concerning since these changes were observed at levels of bilirubin believed to be safe. These findings are being further investigated in an identical ongoing study in premature infants.
